# What type of cell death occurs in chronic cerebral hypoperfusion? A review focusing on pyroptosis and its potential therapeutic implications

**DOI:** 10.3389/fncel.2023.1073511

**Published:** 2023-03-02

**Authors:** Yuxuan He, Xi Chen, Min Wu, Xianhua Hou, Zhenhua Zhou

**Affiliations:** ^1^Department of Neurology, Southwest Hospital, Army Medical University (Third Military Medical University), Chongqing, China; ^2^Department of Neurology, School of Medicine, Chongqing University, Chongqing, China

**Keywords:** pyroptosis, chronic cerebral hypoperfusion, therapeutic implications, inflammasome, inflammatory cytokine, review

## Abstract

Chronic cerebral hypoperfusion (CCH) is a major global disease with chronic cerebral blood flow reduction. It is also the main cause of cognitive impairment and neurodegenerative diseases. Pyroptosis, a novel form of cell death, is characterized by the rupture of the cell membrane and the release of pro-inflammatory mediators. In recent years, an increasing number of studies have identified the involvement of pyroptosis and its mediated inflammatory response in the pathological process of CCH. Therefore, preventing the activation of pyroptosis following CCH is beneficial to inhibit the inflammatory cascade and reduce brain injury. In this review, we discuss the research progress on the relationship between pyroptosis and CCH, in order to provide a reference for research in related fields.

## 1 Introduction

Chronic cerebral hypoperfusion (CCH) is a common pathophysiological process in the central nervous system (CNS) that is caused by chronic cerebral blood flow (CBF) reduction. Several studies have demonstrated that CCH leaves the whole brain tissue in a state of prolonged ischemia and hypoxia, leading to progressive and persistent neurological and cognitive dysfunction, which is related to many cerebrovascular diseases including cerebral white matter demyelination, neurodegeneration, vascular dementia, artherosclerosis, carotid stenosis/occlusion and so on (Starosel’tseva, 2009; Daulatzai, 2017; Duncombe et al., 2017). Compared with acute ischemic stroke, the intervention time window of CCH is longer, and its early diagnosis and treatment has important clinical significance for the prevention and treatment of vascular cognitive dysfunction. CCH can be caused by diseases of large or small cerebral blood vessels such as atherosclerosis or arteriosclerosis in the carotid arteries, which leads to white matter lesions (WMLs), cognitive impairment, and dementia (Bakker et al., 2000; Buratti et al., 2014; Everts et al., 2014; Duncombe et al., 2017). The decrease in CBF due to carotid artery disease leads to cognitive decline (Buratti et al., 2014). Studies have shown that both patients with symptomatic and non-symptomatic carotid artery occlusion exhibit cognitive impairment (Bakker et al., 2000). Cognitive impairment was evaluated in people without vascular stenosis, unilateral carotid stenosis, and bilateral carotid stenosis, and found that the incidence of cognitive impairment increased successively, which was proportional to the degree of vascular stenosis (Buratti et al., 2014). Compared with the normal population, patients with long-term internal carotid artery stenosis have significant impairment in memory, execution, motility, and other aspects, and patients with bilateral internal carotid artery stenosis are more serious (Everts et al., 2014). Therefore, it has a great clinical significance to deeply study the pathogenesis of chronic cerebral ischemia for the prevention and treatment of ischemic cerebrovascular diseases (Yamashita and Abe, 2016).

Current evidences have been demonstrating that CCH could play a critical role in the pathogenesis of vascular contributions to cognitive impairment and dementia (VCID; Román, 2004; Helman and Murphy, 2016). CCH is tightly related to a variety of significant physiological vascular risk factors, as well as VCID pathologies and cognitive decline, all of which suggest that it plays a role in the development of VCID (Qiu et al., 2010; Wolters et al., 2017). VCID has been regarded as a significant vascular pathology process which can lead to vascular dementia and Alzheimer’s disease (AD), both of which are responsible for nearly 60%–80% of dementia cases worldwide (Rizzi et al., 2014; Chang Wong and Chang Chui, 2022). Based on the state of cerebral hypoperfusion observed in VCID patients, various animal models have been used to induce CCH in many previous studies to analyze the underlying pathophysiological mechanisms of VCID (Du et al., 2017; Washida et al., 2019).

Previous studies have identified that the main neuropathological changes of CCH are very complex, including neuronal autophagy, immune inflammatory response, oxidative stress injury, synaptic structural and functional disorders, energy metabolism disorder, cholinergic dysfunction, etc. (Du et al., 2017). Among them, the inflammatory response plays an important role in CCH. It is becoming increasingly clear that the inflammatory process of CCH is the activation of nuclear factor-B (NF-B) and activator protein-1 (AP-1) leads to the release of pro-inflammatory cytokines like tumor necrosis factor (TNF-α) and interleukin-1β (IL-1β), which in turn activates cyclooxygenase-2 (COX-2) and matrix metalloproteinases (MMPs; Rosenberg et al., 2014; Yan et al., 2021). Several studies have illustrated that MMPs were involved in the blood-brain barrier (BBB) disruption of CCH, in which MMP-2, MMP-3, and MMP-9 destroy the basement membrane, tight junctions, and extracellular matrix, increase the permeability of the BBB, and ultimately cause irreversible damage to the BBB following CCH (Ashok et al., 2016; Rosenberg, 2016). In addition, the abnormal changes in glial cells including the proinflammatory phenotype activation of astrocytes and microglia and the loss of oligodendrocytes, play a critical role in the pathological process of the transition from cerebrovascular disease to VCID (Kalaria, 2018). Astrocytes are the most abundant cell type in the CNS, which participate in BBB information, structural support, the regulation of CBF, the maintenance of the neurotransmitters, homeostasis, and the creation of an appropriate extracellular environment (Khakh and Sofroniew, 2015; Rossi, 2015). Astrocytes change their stellate shape to a reactive state in the event of brain injury and degenerative disease. Reactive astrocytes were divided into two types, A1 astrocytes (neurotoxic) significantly upregulate several classical complement cascade genes that are synaptically damaging and A2 astrocytes (neuroprotective) which produce several neurotrophic factors and maintain cerebral homeostasis (Miyamoto et al., 2020). Microglia are crucial innate immune cells that protect the brain and are regarded as the first non-neuronal cells to respond to numerous forms of brain damage (Borst et al., 2021). The typical microglia (M1) phenotype can be activated in the inflammation of cerebrovascular disorders, producing damaging proinflammatory mediators that cause brain tissue destruction (Tang and Le, 2016). On the contrary, M2 phenotype microglia are important for neurogenesis, angiogenesis, and anti-inflammation by generating IL-10 and growth factors such as transforming growth factor β, brain-derived neurotropic factor, and vascular endothelial growth factor (Tang and Le, 2016).

Pyroptosis is a novel form of regulated cell death (RCD) distinct from apoptosis and necrosis, which is triggered by the activation of inflammasomes (Vande Walle and Lamkanfi, 2016). It is manifested that the pyroptosis will undergo a process similar to apoptosis, such as nuclear condensation, DNA fragmentation, and caspase dependence (Chen et al., 2016a). But unlike apoptosis, the pyroptosis is cell membrane swelling and rupture, inducing a violent inflammatory response by the inflammatory caspase 1 or caspase 4/5/11 and releasing pro-inflammatory cell contents. Therefore, pyroptosis is also known as inflammatory apoptosis, which has the partial characteristics of both cell apoptosis and necrosis (Bertheloot et al., 2021). Recently, increasing studies have demonstrated that pyroptosis is involved in the CCH. This review summarizes the molecular mechanisms of pyroptosis and the mechanism of brain injury in CCH briefly and then discusses the current pyroptosis studies in CCH research.

## 2 Molecular mechanism of pyroptosis

In 1992, Zychlinsky et al. (1992) found that gram-negative bacteria could cause the programmed death of host macrophages and the lysis of organelles and cytoplasm under electron microscopy for the first time. This cell death is different from apoptosis that depends on cysteine protease-3 (caspase-3), and mediates host cell death *via* cysteine protease-1 (caspase-1), mediating host cell death with the release of numerous inflammatory cytokines. In 2001, Cookson and Brennan (2001) termed this programmed death as pyroptosis based on their pro-inflammatory characteristics. Next, with the discovery of the caspase downstream substrate gasdermin D (GSDMD; Shi et al., 2015), the understanding of the programmed process of pyroptosis death has become improved. In 2015, several studies found that GSDMD as an important effector protein played a core role in the formation of pyroptosis membrane pores (He et al., 2015; Kayagaki et al., 2015; Shi et al., 2015). In 2019, Liu et al. (2019) further described the three-dimensional structure of human and mouse GSDMD proteins, revealing the inhibitory state of the N-terminus domain and the autoinhibitiory mechanism of gasdermin family proteins, which provided an important structural foundation for the study of the molecular mechanism of GSDMD and caspase binding and the screening of small molecule inhibitors of GSDMD and pyroptosis. GSDMD contains more than 500 amino acids, with N-pore-formingdomain (PFD) and C-repressordomain (RD) distributed at both ends, which can interact to keep GSDMD in an inactive autoinhibitory state (Rogers and Alnemri, 2019; Broz et al., 2020).

**Figure 1:**
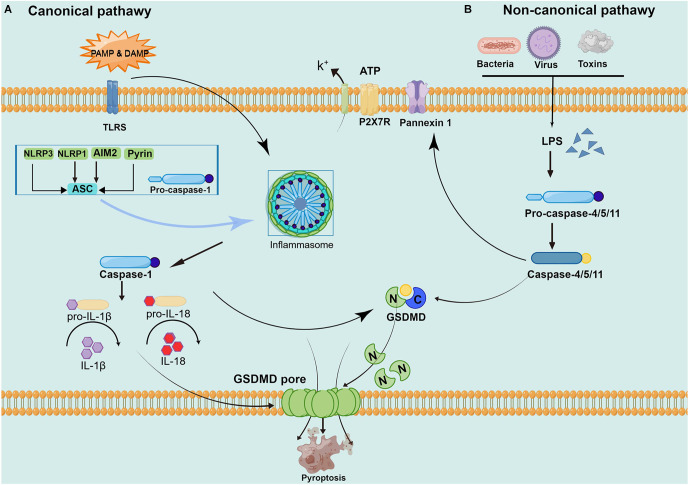
Pathways of pyroptosis activation. **(A) The canonical pyroptosis pathway**: Under PAMP/ DAMP signal stimulation, NOD, ASC, and pro-caspase-1 are successfully assembled into multicomplex inflammasomes. ASC recruits the enzyme ogen caspase-1, which causes automatic cleavage and activation. Activated caspase-1 cleaves inactive pro-IL-1 and proIL-18, turning them into biologically active IL-1 and IL-18, promoting the maturation and secretion of inflammatory cytokines and inducing cell death under inflammatory and stressed pathological conditions. Cell membrane rupture is induced by cleaving GSDMD to form the GSDMD-N and forming pores in the plasma membrane. **(B) The non-canonical pyroptosis pathway**: Stimulated by signals such as LPS, the pro-caspase-11 is activated. On the one hand, activated caspase-11 cleaves GSDMD, forms the GSDMD-N and forms a pyroptosis pore in the plasma membrane, inducing cell membrane rupture and releasing contents. On the other hand, activated caspase-11 induces activated cleaving of caspase-1 to turn pro-IL-1β and pro-IL-18 into IL-1β and IL-18, expanding the inflammatory response and intensifying organismal injury.

Although it also belongs to programmed death, apoptosis has characteristic changes to nucleoplasm concentration, DNA degradation, forming apoptotic bodies, and being engulfed by phagocytes. This process does not produce an inflammatory reaction (Wallach et al., 2014; Galluzzi et al., 2018). Pyroptosis is mediated by caspase-1, and activated caspase-1 cleaves the downstream substrate GSDMD and releases interleukin-1 (IL-1) and IL-18 precursors to produce an inflammatory response (Tsuchiya, 2020). Specific cleavage of GSDMD after caspase-1/4/5/11 activation separates the N-terminus domain and C-terminus domain to release and activate GSDMD-N, and GSDMD-N forms pores containing 16 gasdermin protomers in the cell membrane (Ding et al., 2016). Subsequently, the cells undergo swelling and exocytosis. At this point, intracellular proinflammatory factors (including IL-1, IL-18, etc.) are released and activate a strong inflammatory response. These above studies have revealed that GSDMD, as a unique “porating protein”, is an important executor of pyroptosis (Ding et al., 2016; Kuang et al., 2017; Burdette et al., 2021). However, how GSDMD is recognized by caspase and its association with caspase activation is not completely understood and remains to be further investigated (Wang K. et al., 2020). The main current view is that pyroptosis can be divided into a caspase-1-dependent canonical pathway and a non-canonical pathway dependent caspase-4/5/11 (as shown in Figure 1).

### 2.1 The canonical pyroptosis pathway of caspase-1 dependence

In caspase-1 dependent canonical pyroptosis pathway, activation of caspase-1 exerts its effect through the inflammasome pathway. The inflammasome is a class of multimeric protein complex, to identify a variety of irritating and damaging signals in natural immune responses, which are closely related to the occurrence of cell death (Lamkanfi and Dixit, 2017). In 2002, Martinon et al. (2002) first proposed the concept of the inflammasome, and described the NOD-, LRR- and PYD-containing1 (NLRP1) inflammasome and the nucleotide-binding oligomerization domain-like receptor protein3 (NLRP3) inflammasome. The inflammasome is mainly composed of pattern recognition receptor (PRRs), apoptosis-associated speck-like protein containing CARD (ASC), and pro-caspase-1 (CASP1; Schroder and Tschopp, 2010). PRRs mainly include Toll-like receptors (TLR) family, melanoma deficiency factor 2 (A1M2) family, and NOD-like receptors (NLRs) family, which perform their role by identifying different risk signal molecules and activating the corresponding inflammasomes (Macleod and Bryant, 2017). PRR can identify pathogen-associated molecular patterns (PAMPs) induced by invading pathogens and endogenous pathogen-induced specific molecular patterns (damage-associated molecular patterns, DAMPs). DAMPs is an endogenous substance that is inert and not easily recognized by immune cells under normal conditions. In some inflammatory settings, DAMPs are released after cell death leading to the production of an inflammatory response (Frank and Vince, 2019). ASC is a special adaptor protein, which has two protein domains: pyrin domain (PYD) and CARD. The CASP1 consists of CARD, a large catalytic domain (p20), and a small catalytic subunit domain (p10; Aachoui et al., 2013). ASC recruits the upstream of PRRs and the downstream of pro-Caspase-1 to form the inflammasome. The recruited CASP1 self-catalytic cleaved into two subunits p10 and p20 to form catalytically active Caspase-1, which further cleaves IL-1 and IL-18 precursors to mature IL-1 and IL-18 to mediate pyroptosis.

In conclusion, the canonical pyroptosis pathway of caspase-1 dependence can be summarized as follows. At first, endogenous or exogenous danger signals stimulate inflammasomes after recognition by PRRs through different pathways. Then, the inflammasome forms the inflammasome complex after two stages of priming and activation, which in turn activates its downstream caspase-1. On the one hand, activated caspase-1 either cleaves the target protein GSDMD to the GSDMD-N, forming pores in the cell membrane. On the other hand, catalytically cleaves pro-IL-1 and pro-IL-18 to mature IL-1 and IL-18, released from the cell membrane pore. Therefore, the formation of the inflammasome complex, activation of caspase-1, and cleavage of GSDMD proteins linked and influence in the classical pathway are essential for the occurrence of pyroptosis (Ding et al., 2016; Kovacs and Miao, 2017; Burdette et al., 2021). In animal models of CCH, the activation of NLRP3 and AIM2 inflammasome and activation of the pathway of caspase-1/GSDMD mediated canonical pyroptosis pathway were observed, which was considered the most common cause of neurological impairments (Matsuyama et al., 2020; Poh et al., 2021a, b).

### 2.2 The non-canonical pyroptosis pathway of caspase-4/5/11

In the non-canonical pyroptosis pathway, the caspase recruitment domain (CARD) of caspase-4/5/11 can directly recognize and bind to gram-negative bacterial lipopolysaccharides (LPS) and show obvious protease activity through its own oligomerization reaction (Shi et al., 2014). LPS is recognized by caspase-4/5 in human cells and caspase-11 in mouse cells, which directly makes caspase-4/5/11 active. Then, the active caspase-4/5/11 mediates pyroptosis by cleaving the GSDMD protein and stimulates the pannexin-1pathway. The GSDMD protein is cleaved to GSDMD-N and directly binds to the cellular membrane to form a pore tract that initiates pyroptosis (Man et al., 2017). Pannexin-1 is a channel protein on the cell membrane, which triggers the P2X purinoreceptor 7 (P2X7) receptor-dependent membrane pore opening by mediating the release of ATP, causing K^+^ efflux, osmotic swelling, cell membrane rupture, and eventually leading to pyroptosis (Yang et al., 2015). Caspase-1 is also involved in the non-canonical pyroptosis pathway mediated by caspase-11. As the pannexin-1 transmembrane channel opens, the released ATP can activate the inflammasome NLRP3, the NLRP3 protein and the cohesion protein ASC can directly mediate the activation of caspase-1. While other inflammatory bodies (such as NLRC4 or AIM2) can directly promote the activation of caspase-1, release proinflammatory factors IL-1 and IL-18, and trigger pyroptosis (Schmid-Burgk et al., 2015). Although both caspase-1 and caspase-11 induced pyroptosis, caspase-11 could not directly mediate the maturation of the inflammatory factors IL-1 and IL-18.

In brief, GSDMD achieves caspase enzyme-mediated pyroptosis by punching in the cell membrane. Activation of caspase-1 by the protein complex inflammasome under microbial signals such as viruses and bacteria. Caspase-4/5/11 is activated by direct binding to LPS. Activated caspase-1/4/5/11 cleaves GSDMD to release GSDMD-N and binds to lipid molecules in the plasma membrane to pore in the cell membrane and release proinflammatory factors while changing the osmolarity, the cells gradually swell to membrane lysis and pyroptosis occurs (Yang et al., 2015; Shi et al., 2017). Current evidences have been demonstrating that the non-canonical pyroptosis pathway caspase- 4/11 is also involved in brain injury caused by CCH (Poh et al., 2021b; Moonen et al., 2022).

## 3 Mechanism of brain injury in chronic cerebral hypoperfusion

There are a number of studies have found that the state of CCH can cause vascular endothelial dysfunction, BBB damage, induce oxidative stress and neuroinflammatory response, affect neurotransmitter transmission, make the brain tissue in a state of energy metabolism disorder, cause oligodendrocyte apoptosis, myelin axon damage, white matter damage, synaptic damage, neuronal structure damage, which lead to permanent nerve damage and cognitive dysfunction (Choi et al., 2016; Du et al., 2017; Duncombe et al., 2017).

At present, a variety of CCH animal models have been well established, such as bilateral common carotid artery stenosis (BCAS) mouse, and bilateral common carotid artery occlusion (BCCAO) rats have been widely used in related to CCH mechanism studies (Du et al., 2017; Washida et al., 2019).

### 3.1 The damage of blood-brain barrier

During the process of vascular aging, increased production of reactive oxygen species and inflammatory factors cause excessive oxidative stress and inflammatory responses, stimulate vascular smooth muscle contraction and vascular endothelial cell damage, resulting in decreased cerebral blood flow and increased BBB permeability (Yang et al., 2017). A Meta-analysis showed progressive increases in BBB permeability with age, and the impaired structure and function of BBB are especially obvious in brain injury (Farrall and Wardlaw, 2009). BBB dysfunction could be one of the important mechanisms of brain injury. A study showed that the BBB of BCAS mice observed the close junction opening and vascular endothelial changes at 2 h after surgery (Hattori et al., 2014). A significant reduction of tight junction protein claudin 5 was detected at 6 months after surgery in BCAS mice, with significant disruption of BBB (Holland et al., 2015). The expression of MMP-2 can be detected at 3 days after surgery in BCCAO rats, and BBB is impaired (Sood et al., 2009). MMP-2 plays a key role in the initiation of inflammation in chronic brain hypoperfusion, damaging the basement membrane and tight junction proteins and triggering chronic brain hypoperfusion white matter lesions, resulting in BBB damage, vascular edema, and serum protein extravasation (Tuo et al., 2021). The study proved that MMP-2 inhibition alleviated BBB damage and decreased glial activation and brain white matter damage. And knockdown of MMP-2 caused BCAS mice reduction in the degree of white matter damage and the number of activated glial cells (Seo et al., 2013).

### 3.2 The inflammatory reaction mechanism of chronic cerebral hypoperfusion

The immune system plays a crucial role in maintaining tissue homeostasis as well as in response to infection and injury. Several lines of evidence have demonstrated that CCH as a pathological state will induce neuroinflammation through multiple pathways (Zhang et al., 2020b; Yan et al., 2021; Zhao et al., 2021). The inflammatory response is also one of the significant pathological mechanisms of chronic hypoperfusion-induced brain injury (Wang X.-X. et al., 2020). The harmful substances caused by CCH, especially after the release of necrotic cell debris, can trigger an inflammatory cascade of the immune system, activate microglia and astrocytes (Ben-Ari et al., 2019). Microglia and astrocytes are important components of brain white matter nerve cells, releasing inflammatory mediators to maintain the stability of the nervous system, which are also one of the critical factors leading to brain white matter injury (Fern et al., 2014). The number of microglia and astrocytes in all regions of the white matter increased significantly from 7 days to 14 days after the onset of CCH, and the regions with higher glial cell activity corresponded to the regions with more white matter myelin loss (Shibata et al., 2004). Neuroinflammation produces a cascade leading to ischemic damage and participating in subsequent oxidative damage. Glial cell activation and inflammatory response caused by chronic hypoperfusion are closely related to cognitive dysfunction (Miyanohara et al., 2018; Liu et al., 2021).

#### 3.2.1 Activation of microglial cells

Microglia, the immune cells of the CNS, which is a hallmark of neuroinflammation. The accumulated evidences have shown that microglial activation generally occurs as an early response in cerebral hypoperfusion. After microglia activation, their own structure and function are altered, with a dual role of forming neurotoxicity (M1 microglia) and exerting cerebral protection (M2 microglia; Yang et al., 2014). A previous study has found that numerous M1 microglia proliferate around nerve fiber tangle plaques in the brain of AD patients, causing neuroinflammation (Serrano-Pozo et al., 2016). In a mouse model of transient cerebral ischemia, the M2 microglia response was transitory and phased out within 7 days after cerebral ischemia, and it then progressively changed to the M1 microglia in the peri-infarct region (Hu et al., 2012). However, on the 14th day after BCAS, it was observed that both M1 and M2 microglia were activated in the white matter (Matsuyama et al., 2020). Moreover, Zheng et al. (2022) showed that cornel iridoid glycoside inhibited microglial activation by influencing the microglial M1 to M2 transition in BCCAO rats. At 2 weeks after the onset of chronic cerebral ischemia, the microglial autophagy pathway is initiated, which will induce white matter lesions, induce inflammatory responses, and further aggravate cognitive impairment (Mao et al., 2020). Studies have shown that at the 6th and 8th weeks of chronic cerebral ischemia, microglia activation appeared in the rat hippocampus and cortex area (Tsai et al., 2015). It can be observed that deepening cell staining, enlarged cell body, increased and shorter processes. Other studies have further found that about 13 weeks after CCH in mice, microglia are still activated and 17 weeks after severe chronic brain ischemia, microglial function is impaired, and its amyloid clearance function decreases, which may be related to the pathogenesis of AD (Bordeleau et al., 2016).

#### 3.2.2 Activation of astrocytes

As we all know, astrocytes are the most numerous immune cells in the CNS. Under normal physiological conditions, astrocytes provide metabolic and nutritional support to neurons and maintain their normal function. Reactive astrocytes are diverse, with A1 astrocytes (neurotoxic) and A2 astrocytes (neuroprotective) characteristics. Both A1 and A2 astrocytes are found in the brains of AD patients, but the number of A1 astrocytes is higher than A2 astrocytes, indicating that the preponderance of neuroinflammatory, neurotoxic astrocytes is a key pathogenic hallmark of AD (King et al., 2020). Similarly, in a mouse model of BCAS at 4 weeks, characterized by the number of A1 astrocytes increased, whereas the number of A2 astrocytes decreased (Miyamoto et al., 2020). Studies have shown that after 8 weeks of chronic cerebral ischemia, the number of astrocytes increased significantly, the glial fibrillary acidic protein (GFAP) staining light density increased, and the expression amount increased (Li et al., 2020). This change lasted until 24 weeks later, and the glial scar appeared at the 16th week, which can inhibit axon regeneration and cause irreversible damage (Wang et al., 2016). Alternatively, astrocytes are associated with glutamate metabolism in the brain, and remove glutamate in the synaptic space (Had-Aissouni et al., 2002). At the 10th week after the onset of chronic cerebral ischemia, the number of astrocytes increased further, and the uptake of glutamate in the hippocampus decreased, which may cause cognitive impairment (Vicente et al., 2009).

### 3.3 The injury of brain white matter

As just mentioned above, CCH activates glial cells, and with the increase of BBB permeability, neurotoxic substances such as inflammatory cells enter the brain parenchyma, causing an immune inflammatory response. With the aggravation of neuroinflammatory response, oligodendrocyte progenitor cells are damaged and oligodendrocyte atrophy and die, preventing myelin formation and decreasing myelin density and dissipation, leading to demyelination. The myelinated coated axons also appear serious dysfunction, causing brain white matter damage. At 3 days, glial cell integrity was impaired, and the number of oligodendrocyte precursors and mature oligodendrocytes was reduced in BCAS mice (McQueen et al., 2014). At 14 days, the number of oligodendrocytes in the corpus callosum was significantly decreased and the myelin sheath was dissipated in BCCAO rats (Cai et al., 2011). Demyelination impairs neuronal signaling, causing disruption of linkage pathways in cortical and subcortical regions, and subsequently resulting in alteration and loss of neurocognitive function. White matter damage was observed at 14 days in BCAS mice, severe white matter lose after 30 days, axons and myelin damage in the white matter area, and also seen in the internal capsule, optic tract, and other sites (Shibata et al., 2004).

## 4 Evidence for the cell death occurs and possible pyroptosis-regulated cell death in chronic cerebral hypoperfusion

To date, neuronal death and glial cell death in CCH have been extensively reported. Neuronal loss is a typical characteristic feature observed after CCH. Neuronal death was observed in CCH animal models at 1 week and sustained for up to 3 months following CCH, particularly in the striatum, hippocampus, and cortex, showing that neurons in these three areas are more sensitive to CCH (Ni et al., 1995; Cechetti et al., 2012; Jing et al., 2015). Poh et al. (2021a) reported that neuronal death seems to occur at 15 days and 30 days. However, Guo et al. (2021) showed no difference in the number of neurons by Nissl staining in the cerebral cortex or hippocampus at 30 days after BCAS. Moreover, Liu et al. (2021) reported that there is no change in the number of NeuN-positive neurons in the cortex and hippocampus at 30 days after BCAS. In BCCAO models, apoptotic morphology in hippocampal pyramidal neurons was found at 27 weeks after CCH and further revealed that apoptotic loss of pyramidal neurons may underpin memory impairment related to CCH (Bennett et al., 1998). In addition, the hippocampal atrophy with pyknotic and apoptotic cells was also observed in the brain at 8 months after BCAS (Nishio et al., 2010). In previous reports using an acute cerebral ischemia mouse model, oligodendroglia lose their ultrastructural integrity before neuronal necrosis occurs (Pantoni et al., 1996). Tomimoto et al. (2003) found a significant decrease of oligodendroglia at 14, 30, and 90 days after BCAS with nuclear DNA fragmentation, suggesting that ischemia-induced apoptosis of oligodendrocytes is involved in the pathogenesis of white matter lesions due to CCH. Similarly, the apoptosis of oligodendroglia and myelin disruption in the ischemic white matter was observed 30 days after BCCAO (Cai et al., 2011). Additionally, the increased levels of cleaved caspase-1 were observed in oligodendrocytes of mice at 30 days after BCAS, and further studies revealed that inflammasome-mediated apoptosis and pyroptotic cells death markers were also identified in oligodendrocytes (Poh et al., 2021a). Emerging evidence has reported that the decreased numbers of mature oligodendrocytes and anti-inflammatory microglia were observed at 14 days and 28 days after BCAS with the depletion of regulatory T cells (Wang et al., 2022). These notable findings suggest that neuronal death and glial cell death play critical roles after CCH. In here, we emphasize on the evidence of possible pyroptosis-regulated cell death following CCH, especially in BBB dysfunction, microglia cells, and astrocytes cells, as well as inflammasome mediated cell death in white matter lesion following CCH.

### 4.1 Possible blood-brain barrier dysfunction pyroptosis in CCH

Recent research indicates that pyroptosis contributes to BBB dysfunction. It is widely acknowledged that BBB breakdown can aggravate CCH. Signaling from the inflammasome leads to BBB dysfunction through the activity of IL-1β, which can stimulate the production and release of MMPs from glial cells (Liang et al., 2020). In a rat model of ischemia-reperfusion, a recent study revealed that an interleukin-1 receptor antagonist protected the integrity of the BBB and reduced the production and location of TJPs and MMPs (Zhang et al., 2018). In a transient model of localized cerebral ischemia, both brain-and blood-derived IL-1 are capable of leading to BBB breakdown *via* protein kinase C-theta in human brain microvascular endothelial cells (BMVECs; Rigor et al., 2012; Denes et al., 2013). In animals’ model with ischemic stroke, treatment with IL-1 receptor antagonist (IL-1Ra) or overexpression of IL-1Ra utilizing an adenoviral method significantly reduced BBB injury (Pradillo et al., 2012, 2017). Caspase-1 was found to induce BBB dysfunction *via* the induction of pyroptosis in a comparable scenario where its enzymatic activity was inhibited (Liang et al., 2020). Moreover, several studies have indicated that the endothelial NLRP3 inflammasome may be involved in controlling the BBB in a range of illnesses (Chen et al., 2016b; Lian et al., 2019; Zhang et al., 2019; Bai et al., 2020), yet there is still no concrete proof of its involvement in CCH. *in vitro* and *in vivo* evidence from research on ischemic brain damage suggests that the NLRP3 inflammasome may be involved in the breakdown of the BBB in CCH (Hou et al., 2018). The NLRP3 inflammasome has a major role in the BBB disruption caused by ischemic stroke, triggering inflammatory pathways and causing pyroptosis in brain endothelial cells (Bellut et al., 2021). In addition, the NLRP3 inflammasome inhibitor MCC950 suppressed the inflammatory response of BBB disruption caused by ischemic stroke (Bellut et al., 2021). Although these findings may account for the BBB disruption that is observed in CCH, more studies are still needed to verify it in the future.

### 4.2 Possible microglia pyroptosis in CCH

Current research evidence suggests that the activation of microglia pyroptosis is a significant factor in neuroinflammation and is linked to several neurological diseases, including ischemic brain injury, and neurodegenerative diseases (Wu et al., 2022). Research has discovered that the expression of NLRP3 in microglia of patients with ischemic stroke is increased, and the proteins involved in NLRP3 inflammasomes, as well as IL-1β and IL-18, are also expressed at higher levels in the mouse model of MCAO/R (Fann et al., 2013). In their resting state, microglial cells are equipped with many PRRs that screen the microenvironment (Prinz et al., 2019). Under certain pathological conditions, such as LPS, microglia are rapidly stimulated and produce a wide range of proinflammatory substances in the CNS, resulting in a highly inflammatory state (Harry, 2013; Zhang et al., 2022). A previous study had revealed that IL-1α and IL-1β were produced as a result of NLRP3 inflammasome activation *via* DAMPs in acute cerebral ischemia models, which regulated glial cell inflammatory responses and induce neuroinflammation in the CNS (Savage et al., 2012). Many studies have revealed that microglia may be responsible for both inflammatory and non-inflammatory reactions through their interaction with NLRP3 inflammasome and AIM2 inflammasome during CCH (Su et al., 2019; Poh et al., 2021a). In addition, a previous study has indicated that CCH can increase amyloid-β deposition in the intracellular compartment as well as NLRP3, activated caspase-1 and IL-1β expressions in the hippocampus and thalamus, potentially contributing to the development of AD (Shang et al., 2019). Moreover, the progression of AD is characterized by the swift binding of CARD/ASC-containing bridging proteins released by microglia pyroptosis to Aβ, which leads to an increase in Aβ oligomer and aggregate formation (Venegas et al., 2017). However, no study to date has directly investigated microglia pyroptosis following CCH.

### 4.3 Possible astrocytes pyroptosis in CCH

The mounting evidence suggests that the inflammatory responses triggered by astrocyte pyroptosis may be a contributing factor to brain injury (Kim et al., 2020; Jiang et al., 2021), and related neuroinflammation appears to play a role in the pathology of neurological dysfunction caused by CCH (Voet et al., 2019). In BCAS models, researchers have directly observed that the expression of the NLRP3 inflammasome was increased in astrocytes (Matsuyama et al., 2020). And the NLRP3 inflammasome is involved in astrocyte dysfunction (Johann et al., 2015; Zhu and Tang, 2020) and the inflammatory response following CCH (Shang et al., 2019). A recent study on AD patients reported the presence of caspase-8 in astrocytes which may indicate an alternative pathway for GSDMD cleavage and activation of pyroptosis (Moonen et al., 2022). Moreover, astrocytes contribute to injury CNS related to neuroinflammation through the release of proinflammatory cytokines and toxic molecules, which leads to a negative impact on neuronal function and can lead to severe neurological consequences (Choudhury and Ding, 2016; Sharma et al., 2016). As the astrocytes undergo pyroptosis, the astrocytes swell, compress vessels, and further aggravate the vascular hypoperfusion (Zheng Z. et al., 2021). After oxygen-glucose deprivation/reoxygenation (OGD/R) in astrocytes, NLRP6 and its activation product generation increased (Zhang et al., 2020a). Furthermore, NLRP6 interacted with ASC, which decreased the release of inflammatory cytokines and increased neuronal viability (Zhang et al., 2020a). Hence, it seems that astrocytes pyroptosis may be possibly involved in CCH-induced brain injury.

### 4.4 Inflammasome mediated cell death in white matter lesion following CCH

CCH induces white matter lesions and hippocampus atrophy and is one of the most important factors in VCID (Román, 2004; Helman and Murphy, 2016). Some experimental results from a chronic hypoperfusion-induced mouse VCID model demonstrated that gliosis and a prolonged inflammatory response play a key role in white matter lesion formation (Chen et al., 2017). Yoshizaki et al. (2008) discovered that C57BL6/J mice with CCH have elevated levels of pro-inflammatory cytokines IL-1β and IL-6 and decreased levels of anti-inflammatory cytokines IL-4 and IL-10 in the corpus callosum and developing white matter lesions. At 30 days after BCAS, Liu et al. (2021) identified significant activation of glial cells such as astrocytes and microglia in the corpus callosum, external capsule, and hippocampus, which was related with demyelination and microstructural damage in the white matter and hippocampi. Moreover, several studies have shown that a tight relationship between inflammasome activity and the production of white matter lesions, which is commonly accompanied by activated glia (Simpson et al., 2007; Waller et al., 2019). Glial activation was found in patients with AD at an early stage, and it was hypothesized that the ensuing glia-mediated inflammatory process was responsible for the advancement of cognitive impairment (Nordengen et al., 2019). The available information indicates that the inflammasome signaling pathway more likely plays a causal role upstream of CCH-induced white matter lesions formation.

## 5 The mechanisms of pyroptosis in the pathogenesis of chronic cerebral hypoperfusion

**Figure 2:**
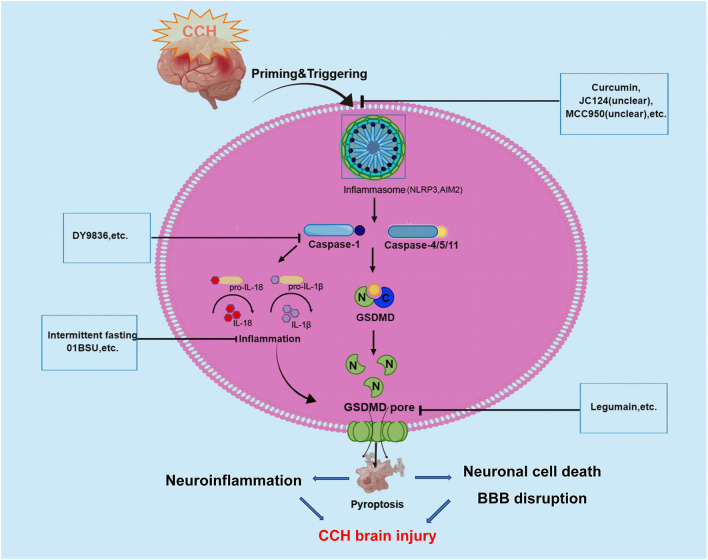
Graphical description of pathological mechanism of pyroptosis following CCH brain injury and potential drugs targeting pyroptosis-regulated cell death in CCH. During the onset of CCH, NLRP3, and AIM2 inflammasome are activated and then stimulate caspase-1/4/5/11. Caspases-1 and -4/5/11 are both responsible for the processing of GSDMD, which leads to the release of the GSDMD-NT. The formation of pores by GSDMD-NT in the plasma membrane and organelle membranes is what causes pyroptosis to occur. In the case of the pores in the plasma membranes of endothelium and neuronal cells directly could cause cell lysis, which induces BBB disruption and neuronal cell death. At the same time, inflammatory cytokines are released *via* pores and contribute to the regulation of neuroinflammation. These underlying pathophysiological processes will aggravate CCH-related brain injury. To date, several inhibitors of NLRP3, AIM2 inflammasome, Caspase-1, and GSDMD have been described for promising in alleviating CCH-related brain injury.

As we all know, CCH is a pathological state caused by long-term cerebral blood perfusion, which is the pathological basis of various neurological diseases such as vascular dementia VCID, AD, and Binswanger disease. Although the pathogenesis of CCH is not fully understood a key factor underlying the pathogenesis of chronic cerebral ischemic injury may be an inflammatory response. Recently, increasing studies have demonstrated that the inflammatory responses after CCH in the brain could be triggered by innate immune multiprotein complexes which were regarded as inflammasomes. Inflammasomes trigger pyroptosis through the activation of caspase-1 to mediate the classical inflammasome pathway and the caspase-11/4/5-mediated non-canonical inflammasome pathway. And pyroptosis plays a crucial role in the regulation of inflammation, so there could be a close link between pyroptosis and CCH. Caspase-1 protease is a core component of the polyprotein inflammasome complex, which can promote the release of a large number of proinflammatory factors such as IL-1 and IL-18, participating in the intrinsic inflammatory immune response (as shown in Figure 2).

In recent years, increasing evidence has shown that CCH can activate the inflammasome signaling pathways, involving NLRP3 and AIM2 inflammasomes that critically regulate IL-1β production (Matsuyama et al., 2020). Several studies have revealed that CCH induced a complex temporal expression and activation of the AIM2 inflammasome and their downstream products (IL-1β and IL-18) in cortical and hippocampal neurons, and promotes activation of pyroptosis (Poh et al., 2021a). In a BCAS mouse model, Poh et al. (2021a) observed the inflammasomes in the cerebral cortex, hippocampus, and striatum following CCH, and found increased expression and activation of NLRP3 and AIM2 inflammasome receptors, especially in the hippocampal region (Matsuyama et al., 2020). Further assays demonstrated that AIM2 inflammasome activation mediates apoptosis and pyroptosis of cortical and hippocampal neurons following CCH. Then, the same authors found emerging evidence suggesting that AIM2 inflammasome mediates increased release of the proinflammatory cytokines IL-1β, further activating cell pyroptosis in the cerebellum during CCH (Poh et al., 2021b). In their study, increased non-canonical inflammasome activation was also observed, with higher levels of cleaved caspase-11 in the BCAS model at 21 days (Poh et al., 2021b). Notably, previous studies have shown that non-canonical inflammasome protein caspase-4 was involved in the AD pathogenesis process and Aβ-induced cell death (Hitomi et al., 2004; Nishizaki, 2019). A recent study reported that caspase-4 in cleaved GSDMD-positive neurons were presented in the brains of AD patients may indicate non-canonical inflammasome-mediated pyroptosis and cleavage of GSDMD (Moonen et al., 2022). Besides, Matsuyama et al. (2020) reported that CCH induced upregulation of NLRP3 and AIM2 inflammasomes in the white matter and corpus callosum of BCAS mice model. This study further observed that the expression of NLRP3 inflammasome in astrocytes and AIM2 inflammasome in microglia were increased in the autopsied cerebral white matter lesion of patients with cerebral infarction in the chronic phase. In a right unilateral common carotid artery occlusion (rUCCAO) mice model, Chai et al. (2021) showed that the expression of pyroptosis effector protein GSDMD was obviously increased and the density of neurons in the dentate gyrus (DG) region declined. The results revealed that GSDMD could play a key role in the occurrence of pyroptosis following CCH.

All these studies indicated that pyroptosis may be involved in brain injury following CCH through the canonical pyroptosis pathway of NLRP3/AIM2/Caspase-1/GSDMD and non-canonical pyroptosis pathway of caspase-4/11.

## 6 The potential therapeutic implications of pyroptosis inhibitors in chronic cerebral hypoperfusion

Due to the critical role of inflammasome-mediated pyroptosis in the neuroinflammatory response and neural tissue injury after CCH, therapeutic agents targeting to inhibit inflammasome-mediated pyroptosis have been extensively studied. It has great significance for us to explore mechanisms and clinical treatment in the future. Here, we summarize the drugs currently known to have an effect on pyroptosis following CCH as well as potential drugs that may be therapeutic targets for CCH (as shown in Table 1).

**Table 1:** Summary of potential agents or targets suppressing pyroptosis and inflammasome components to treat chronic cerebral hypoperfusion.

Agents	Targets	Model	Mechanisms of pyroptosis and inflammasomes inhibition	References
Curcumin		BCCAO rats	NLRP3/ASC/Cleaved-caspase-1/GSDMD-N/IL-18	Zheng Y. et al. (2021)
DY9836		BCAS mouse	NLRP3/Caspase-1/IL-1β	Wang et al. (2017)
IL-1β antibody (01BSU)		ACAS	IL-1β	Quintana et al. (2021)
	AIM2 inflammaome inhibitors	AIM2 knockout BCAS mice	Cleaved caspase-1/Cleaved caspase-11/IL-1β/GSDMD-NT	Poh et al. (2021b)
	Intermittent fasting	BCAS mouse	Cleaved caspases-1 (p33), −8, −11/IL-18/IL-1β	Poh et al. (2021c)
	Legumain	rUCCAO mice	GSDMD/P65	Chai et al. (2021)

### 6.1 Native compound

Curcumin is a natural polyphenol extracted from the rhizome of curcuma longa Linn, which protects neurons from damage and has strong anti-inflammatory, anti-oxidant, and anti-tumoractivities (Catanzaro et al., 2018; Kahkhaie et al., 2019). A recent study has found that curcumin could alleviate neuroinflammation by inhibiting the activation of microglial *via* regulating the triggering receptor expressed on myeloid cells 2 (TREM2)/TLR4/NF-κB signaling pathways (Zheng Y. et al., 2021). Moreover, curcumin could further alleviate CCH-induced apoptosis, and reduce nod-like receptor protein 3(NLRP3)-dependent pyroptosis, thereby improving secondary brain damage and behavioral disorders after CCH (Zheng Y. et al., 2021).

### 6.2 Calmodulin inhibitor

As a calmodulin inhibitor, DY9836 is derived from DY9760e, which can preserve the BBB integrity during cerebral edema caused by microsphere embolism (Han et al., 2006; Lu et al., 2009). In the BCAS model, DY-9836 may significantly inhibit the expression and assembly of NLRP3 by inhibiting protein tyrosine nitration, caspase-1, and IL-1β production, and reduce oxidative stress, which will reduce the damage of cerebral cortical and enhance the cognitive function (Wang et al., 2017). However, it indirectly or nonspecifically prevents NLRP3 activation and, when translated into clinical practice, may lead to a higher incidence of adverse effects.

### 6.3 Specific inflammasome inhibitors

In AIM2 knockout BCAS mice model, Poh et al. (2021a) observed that the inflammasome-mediated production of proinflammatory cytokines, apoptosis, and pyroptosis. Therefore, the AIM2 inflammasome inhibitors may provide a promising therapeutic target for CCH. Glemepiride, a known anti-diabetic drug (sulfonylurea), has been shown to inhibit the ATP enzyme activity of NLRP3, but its high dose required to inhibit NLRP3 *in vivo* causes lethal hypoglycemia (Lamkanfi et al., 2009). Therefore, it cannot be directly used as an NLRP3 inhibitor. The JC124 was developed by optimizing the structure of glimepiride. JC124 was a novel NLRP3-specific inhibitor that eliminated the potential hypoglycemic effect of glimepiride and improved the selectivity of NLRP3 (Fulp et al., 2018). Therefore, it could significantly reduce the common adverse effects of anti-inflammatory molecules. Besides, MCC950 also known as cytokine release suppressor 3 (CRID3), was the most potent and selective NLRP3 inhibitor (Coll et al., 2019). It was also derived from antidiabetic drugs, and not inhibiting NLRP1, NLRC4, and AIM2 inflammasome, showing neuroprotective effects in stroke and cerebral hemorrhage models (Ren et al., 2018; Zhu et al., 2021). MCC950 attenuates NLRP3 activation and subsequent mature L-1 secretion, reduces brain edema, tissue loss, microglial activation, leukocyte infiltration, and BBB destruction, and improves neural functions such as learning and memory after stroke (Zhu et al., 2021). However, there are few studies reporting that the role of specific inflammasome inhibitors such as JC124 and MCC950 in CCH and needs to be further determined. Interestingly, Poh et al. (2021c) further found that intermittent fasting could eliminate inflammasome-associated apoptosis and pyroptosis in the brain following CCH in a BCAS mouse model.

### 6.4 Inhibitors of the inflammasome-related molecules

Current studies have reported that IL-1β antibody was clinically used in a variety of autoimmune and inflammatory diseases. IL-1β is also one of the major inflammatory cytokines implicated in the pathogenesis of CCH. More recently, Quintana et al. (2021) first revealed that IL-1β antibody (01BSU), a highly specific IL-1β monoclonal antibody, could significantly reduce the damaged volume of the brain and protects the brain from CCH in the mice using ameroid constrictor arterial stenosis (ACAS) surgery model. Moreover, Chai et al. (2021) have confirmed that the involvement of legumain knockout improved cognitive impairment by reducing neuroinflammation and impeding the activation of P65 and pyroptosis in rUCCAO mice. It also indicated that legumain would be a potential novel target in the future for CCH treatment with great significant (Chai et al., 2021).

Together, these studies provide potential for the inflammasome-related molecules inhibiting targets that could suppress pyroptosis in CCH.

## 7 Conclusions and future perspectives

Pyroptosis is a proinflammatory form of programmed cell death characterized by cell membrane rupture and release of inflammatory mediators. Inflammasome, caspase-1/4/5/11, and GSDMD participate in the process of cell pyroptosis through the canonical pyroptosis pathway and non-canonical pyroptosis pathway, respectively. GSDMD is a key effector protein discovered in recent years, which mediates the occurrence of cell pyroptosis by forming pores on the cell membrane, causing osmotic swelling and releasing inflammatory factors. Pyroptosis has an important role in the development of CCH and can be reduced by regulating the level of key pyroptosis factors. At present, targeted drugs for key proteins in the pyroptosis pathway have been proven to reduce chronic ischemic brain injury to some extent, but theirresearch is mainly limited to the cell and animal experimental level. Therefore, the existing research results can be improved to further strengthen the clinical research on the relationship between pyroptosis-related procesess and CCH, so as to provide more effective strategies for the treatment of CCH.

## Author contributions

XH and ZZ are joint corresponding authors. YH, XH, and ZZ contributed to the conceptualization of the manuscript. YH, XC, MW, XH, and ZZ contributed to the writing of the manuscript, contributed to the editing and revising of the manuscript. All authors contributed to the article and approved the submitted version.

## Funding

This work was supported by the National Natural Science Foundation of China (nos. 81971130, 81471194, and 81601025), the General program of Chongqing Natural Science Foundation (cstc 2020 jcyj-msxmX1099), and Talents Training Program of Army Medical University (XZ-2019-505-074).

## Conflict of Interest

The authors declare that the research was conducted in the absence of any commercial or financial relationships that could be construed as a potential conflict of interest.

## Publisher’s Note

All claims expressed in this article are solely those of the authors and do not necessarily represent those of their affiliated organizations, or those of the publisher, the editors and the reviewers. Any product that may be evaluated in this article, or claim that may be made by its manufacturer, is not guaranteed or endorsed by the publisher.
